# Cross-lagged analysis of burnout and insomnia in older adult patients with diabetes mellitus complicated by hypertension: a 1-year longitudinal follow-up study

**DOI:** 10.3389/fpubh.2025.1662118

**Published:** 2025-09-09

**Authors:** Bailing Zhang, Fengfei Zhou, Weipeng Qian, Qingqing Yang

**Affiliations:** ^1^Department of Cardiology, The Sixth People's Hospital of Nantong, Nantong, Jiangsu, China; ^2^Department of Endocrinology, The Sixth People's Hospital of Nantong, Nantong, Jiangsu, China

**Keywords:** older, diabetes, hypertension, burnout, insomnia, cross-lagged study

## Abstract

**Objective:**

The aim of this study is to investigate the longitudinal bidirectional relationship between disease management burnout and insomnia symptoms in older adult patients with diabetes mellitus complicated by hypertension.

**Methods:**

A prospective cohort study was conducted, involving 326 older adult patients from the Sixth People’s Hospital of Nantong City, enrolled between January 2023 and April 2025. The study utilized the Burnout in Disease Management Scale and the Insomnia Severity Index (ISI) to perform four-stage evaluations at baseline (T0), 3 months post-discharge (T1), 6 months post-discharge (T2), and 12 months post-discharge (T3). A cross-lagged structural equation model was employed to analyze the bidirectional relationship between burnout and insomnia, while accounting for confounding factors such as gender and comorbidities.

**Results:**

The influence of burnout on insomnia was significant, with burnout at T0 strongly predicting insomnia at T1 (*β* = 0.29, *p* < 0.001), and the predictive effect of burnout at T2 on insomnia at T3 peaking (*β* = 0.24, *p* < 0.001). Conversely, the impact of insomnia on burnout was also evident, with insomnia at T0 predicting burnout at T1 (*β* = 0.15, *p* < 0.05), insomnia at T1 predicting burnout at T2 (*β* = 0.19, *p* < 0.01), and insomnia at T2 predicting burnout at T3 (*β* = 0.21, *p* < 0.01). Additionally, a gender moderation effect was observed, with women exhibiting a higher degree of burnout at baseline (*β* = −0.11, *p* < 0.05), and the predictive effect of burnout at T2 on insomnia at T3 was more pronounced in women.

**Conclusion:**

A significant bidirectional and vicious cycle relationship exists between disease management burnout and insomnia symptoms in older adult patients with diabetes and hypertension. Furthermore, T2 (6 months post-discharge) emerges as a critical window for intervention. It is recommended that gender-differentiated intervention strategies be implemented and that sleep management be integrated into the standardized diagnosis and treatment pathway for this patient population.

## 1 Introduction

As the pace of life accelerates and the population ages, insomnia has emerged as a global public health challenge. Data indicates that the prevalence of insomnia is increasing worldwide, significantly impacting individuals’ physical and mental health and quality of life (1). Sleep issues are particularly acute in China, where the “China Sleep Quality Survey Report” reveals that over 83% of respondents frequently experience sleep problems (2). Notably, older adults, a demographic with a high incidence of insomnia, face more complex and severe sleep issues (3). The decline in physiological function, increased chronic disease burden, and changes in social roles associated with aging are key factors contributing to older adults’ insomnia. Among various chronic diseases, diabetes mellitus (DM) and hypertension (HTN) are prevalent and often coexist in older adults (4). Studies have demonstrated that the incidence of insomnia in older adult patients with both DM and HTN is markedly higher than in healthy individuals, reaching as much as 44.8 to 62.5% (5, 6). The presence of multiple chronic diseases further exacerbates the risk of sleep disorders. Insomnia not only directly harms the physical and mental well-being of patients but also creates a vicious cycle by reducing treatment compliance, disrupting blood glucose and blood pressure control, and elevating the risk of cardiovascular and cerebrovascular complications and mortality. Consequently, it is imperative to investigate the influencing factors and mechanisms of insomnia in older adult patients with DM and HTN to enhance their health outcomes.

The primary symptoms of insomnia in older adult patients with DM and HTN include difficulty initiating sleep, sleep maintenance issues (such as frequent nighttime awakenings), early morning awakenings, and a significant reduction in sleep quality (7). These chronic sleep disturbances not only result in daytime fatigue, lack of concentration, and cognitive decline (8), but also intensify negative emotional experiences (such as anxiety and depression) (9), severely impacting their ability to manage disease challenges and daily life. In recent years, the concept of “Patient Burnout” within the realm of chronic disease management has garnered increasing interest. Drawing from the theory of job burnout, patient burnout is characterized as the physical and mental exhaustion experienced by patients with chronic diseases during the long-term self-management of their condition (10). This burnout is especially pronounced in older adults patients with DM and HTN, and is marked by Emotional Exhaustion: Faced with complex treatment regimens, including multiple medications, blood glucose and blood pressure monitoring, dietary and exercise control, frequent medical appointments, financial strain, and the uncertainty of their disease, patients experience profound feelings of helplessness, fatigue, and negative emotions, feeling “hollowed-out” by the disease. To personal/alienation (Depersonalization/Cynicism): Patients’ disease management begins to become “cold” or “absent, “adopting an evasive attitude, actively disengaging from positive self-management behaviors, as long as the symptoms are not severe or life-threatening. Reduced Personal Accomplishment: The patient loses confidence in their ability to control the disease, believes that no matter how much effort is invested in improving their health condition, they fall into a state of helplessness and despair.

Although a large number of cross-sectional studies have confirmed the significant association between burnout level and insomnia in older adult patients with chronic diseases (11, 12), the existing studies have obvious limitations. The direction of causality is unclear, and the cross-sectional design cannot distinguish whether the long-term burden of disease management triggers burnout and leads to insomnia, whether insomnia exacerbates burnout through decreased energy and cognitive-emotional functioning, or both create a vicious cycle. There is a lack of longitudinal evidence for older adults with multimorbidity, especially for older adults who are facing the dual burden of disease management of DM and HTN. Longitudinal studies to explore the dynamic interaction between burnout and insomnia are extremely scarce. There is insufficient in-depth research on the application of the concept of burnout, particularly the analysis of its mechanism of action with insomnia based on the dimensions of burnout (fatigue, alienation, sense of control).

Therefore, to clarify the temporal relationship and possible bidirectional influence mechanism between disease management burnout and insomnia symptoms in older adult patients with DM and HTN, this study adopted a prospective longitudinal design with a 1-year follow-up. Using a Cross-Lagged Panel Analysis (CLPA) model, it examined the relationship between disease management burnout and insomnia symptoms. Hypothesis H1: Baseline burnout can significantly and negatively predict the severity of insomnia 1 year later. Hypothesis H2: Insomnia severity at baseline can significantly and negatively predict burnout 1 year later. The results of this study will provide an important basis for understanding the deep psychological motivations of sleep disorders in this population, and provide key scientific support for the future targeted development of integrated intervention strategies to reduce burnout or improve sleep as the entry point, so as to interrupt the vicious cycle and ultimately improve the overall health management and quality of life of patients.

## 2 Subjects and methods

### 2.1 Subjects

#### 2.1.1 Calculation of sample size

The study used a prospective longitudinal design, and the sample size was comprehensively estimated with reference to the following criteria:

1. Variable proportion method of structural equation model (13), based on 19 observed variables (12 items of disease management burnout Scale + 7 items of insomnia index scale), calculated as *N* = 15 × number of variables, 285 cases were needed, and 320 cases were set up after 10% redundancy.
2. Compensation Method for Loss to Follow-Up: Utilizing the follow-up data for chronic diseases from the Cardiology/Endocrinology Department of our hospital, which has a loss rate of 20–25%, we initially recruited 340 patients to ensure that 285 patients completed the 4-point follow-up schedule (at discharge, 3 months, 6 months, and 12 months).
3. Statistical Power: The G*Power 3.1 calculation indicated that a moderate effect size (*β* ≥ 0.30) required more than 246 participants (*α* = 0.05, power = 0.90). The final inclusion plan was to enroll 350 patients upon discharge.

#### 2.1.2 Inclusion and exclusion criteria

The study utilized a convenience sampling method. Between January 2023 and April 2025, the Department of Cardiology and Endocrinology at the Sixth People’s Hospital of Nantong selected 350 cases of diabetic patients with hypertension. The inclusion criteria were as follows: ① age of 60 years or older; ② diagnosis aligning with the type 2 diabetes diagnostic criteria outlined in the “Chinese Guidelines for the Prevention and Treatment of Type 2 Diabetes” (14); ③ diagnosis consistent with the essential hypertension criteria specified in the “Chinese Guidelines for the Prevention and Treatment of Hypertension” (15); ④ patients diagnosed and receiving treatment for over 6 months, scheduled for regular follow-up at our hospital; ⑤ conscious status with an MMSE score >20 (16); and ⑥ permanent residence in Nantong City for a minimum of 1 year. The exclusion criteria were as follows: ① presence of organic sleep disorders such as sleep apnea syndrome or restless legs syndrome; ② use of sleep-promoting medications, such as benzodiazepines, two or more times per week during the month prior to baseline assessment; ③ interventions that could disrupt sleep patterns, such as nocturnal dialysis or continuous insulin pump therapy; ④ presence of advanced malignant tumors or NYHA class IV heart failure or eGFR <30 mL/min/1.73 m^2^ (CKD stage 4–5); and ⑤ life expectancy less than 15 months as assessed by the attending physician. Written informed consent was obtained from all participants. This study was approved by the Ethics Committee of the Sixth People’s Hospital of Nantong (NTLYLL2023009).

### 2.2 Research tools

#### 2.2.1 General information questionnaire

This questionnaire comprises three sections: demographic characteristics, disease characteristics, and treatment burden. (1) Demographic characteristics include age, gender, living situation, educational level, and occupation prior to retirement; (2) Disease characteristics encompass the duration of diabetes, the duration of hypertension, glycated hemoglobin levels, and the number of concurrent complications; (3) Treatment burden covers the number of daily medications, daily injection volume, and self-monitoring requirements.

#### 2.2.2 Disease management burnout scale

This study employed the Chronic Disease Patient Burnout Scale, created by Abdoli et al. (17), which was methodically translated into Chinese and culturally tailored for use among the older adults in China. The scale consists of 12 items across three dimensions: exhaustion (4 items), which assesses the physical and mental exhaustion resulting from disease management; disengagement (5 items), which evaluates the tendency to detach and avoid disease management; and lack of control (3 items), which reflects feelings of powerlessness and diminished confidence in managing the disease. Responses were scored using a 5-point Likert scale (1 = strongly disagree, 5 = strongly agree). Dimension scores were calculated as the average of the items within each dimension (ranging from 1 to 5), and the total score was the sum of all item scores (ranging from 12 to 60). A higher total score indicates a more severe overall level of burnout. The pretest phase of this study (n = 30) demonstrated good reliability and validity for the scale, with a total Cronbach’s *α* of 0.89 (exhaustion 0.84/disengagement 0.79/lack of control 0.76), and confirmatory factor analysis confirmed the three-factor structure (CFI = 0.93, RMSEA = 0.07).

#### 2.2.3 Insomnia severity index

Seven items were used to evaluate various aspects of sleep difficulty, including initial insomnia, maintaining sleep, early awakening, satisfaction with sleep quality, daytime dysfunction, visible sleep problems, and the degree of insomnia-related distress (18). A 5-point scale was implemented, ranging from 0 (none) to 4 (extremely severe), resulting in a total score between 0 and 28 points. The clinical classification was as follows: 0 to 7 points indicated no insomnia; 8 to 14 points, mild insomnia; 15 to 21 points, moderate insomnia; and 22 to 28 points, severe insomnia. Insomnia was considered positive when the total score reached or exceeded 8 points. The internal consistency was high, with a Cronbach’s *α* of 0.80. Structural validity was confirmed through confirmatory factor analysis, which supported a two-factor model (symptom dimension and function dimension), explaining a cumulative variance of 63.1%.

### 2.3 Data collection methods

This study employed a standardized procedure to collect data at four distinct time points: T0 (48 h prior to discharge), T1 (3 months ± 5 days), T2 (6 months ± 7 days), and T3 (12 months ± 10 days). At T0, face-to-face interviews were conducted in a private room within the ward by trained nurses. Structured assessments were performed, which included the verbal administration of the 12-item Burnout Scale and the completion of the 7-item ISI Scale on an electronic tablet. Concurrently, physiological indicators (such as HbA1c, blood pressure, and medication regimens) were retrieved from the medical record system. For T1 and T2, a mixed-mode approach was utilized, with the primary method being standardized telephone follow-ups (with double-blind entry of scale data). At T3, a multimodal approach was implemented, with a preference for outpatient face-to-face interviews (similar to T0). For individuals with mobility constraints, home visits or video verification (with screen sharing to document the process) were arranged. To verify consistency, 20% of the samples were re-evaluated through phone calls (Kappa ≥ 0.85). The entire process required nurses to maintain an ICC consistency of ≥ 0.90, and community collaboration was employed to track participants lost to follow-up. Participants who completed at least three assessments were included in the ITT analysis set. The significance of data collection at these specific time points is detailed in Table 1.

**Table 1:** Time point settings and clinical significance.

Evaluation time point	Time	Time selection criteria
T0 (Baseline)	48 h prior to discharge	Mark the patient’s entry into the stable period, manage and control acute inpatient disturbances, and document the initial levels of fatigue and insomnia.
T1	T0 + 3 months ± 5 days	During the stable adjustment period of the drug regimen, observe the short-term adaptation of sleep to stress management.
T2	T0 + 6 months ± 7 days	The six-month disease control assessment node (HbA1c monitoring cycle) identifies the bidirectional interaction between the accumulation of burnout and sleep problems.
T3	T0 + 12 months ± 10 days	Evaluate the long-term management outcomes, including annual complication screenings, verify the cross-lagged effects, and ensure that the model adheres to the temporal sequence requirements.

### 2.4 Statistical approach

Data preprocessing and preliminary statistical analyses were conducted using SPSS 27.0. The Shapiro–Wilk test was employed to assess the normality of the distributions. The total burnout score, its subscales, and the ISI scores across time points T0 to T3 all followed a normal distribution (*p* > 0.05). Descriptive statistics were computed and expressed as means along with standard deviations. Pearson correlation coefficient matrices were generated to illustrate both concurrent and longitudinal associations among variables. In the main analytical phase, Mplus 8.7 was utilized to develop a cross-lagged structural equation model across four measurement occasions. The analytical procedure was structured as follows: First, measurement invariance over time was evaluated through multi-group confirmatory factor analysis to examine the longitudinal equivalence of the burnout inventory, which comprises three dimensions—fatigue, disengagement, and loss of control—across the four time points. The criteria for acceptable measurement invariance included ΔCFI ≤ − 0.010, ΔRMSEA ≤ 0.015, and ΔSRMR ≤ 0.030. A latent variable model was then specified, incorporating four waves of latent constructs representing burnout and insomnia at T0, T1, T2, and T3. This model estimated three key types of paths: autoregressive paths to assess the temporal stability of burnout and insomnia, cross-lagged paths to evaluate reciprocal influences between the two constructs, and contemporaneous correlations between residuals of burnout and insomnia at each time point. For parameter estimation and handling of missing data, robust maximum likelihood estimation (MLR) was applied to mitigate the influence of potential outliers, while the full information maximum likelihood (FIML) approach was used to manage missing values under the assumption of missing completely at random. Model fit was evaluated based on conventional thresholds: acceptable fit was indicated by CFI > 0.90, TLI > 0.90, RMSEA < 0.08, and an excellent fit by CFI > 0.95, TLI > 0.95, RMSEA < 0.06.

## 3 Result

### 3.1 Control of common method bias

To assess potential bias in longitudinal data, this study employed a dual-method verification approach, specifically Harman’s single-factor test. Exploratory factor analysis was conducted on the combined scale items from the four time points (T0-T3), which included 12 items for burnout and 7 items for insomnia, totaling 76 items. The KMO sampling adequacy coefficient was 0.82, and the Bartlett’s sphericity test yielded a chi-square value of 5327.36 (*p* < 0.001). Eleven factors with eigenvalues >greater than 1 were extracted, and the maximum factor variance contribution rate was 31.7%, indicating that common method bias was not severe.

### 3.2 General characteristics of the research subjects

**Figure 1:**
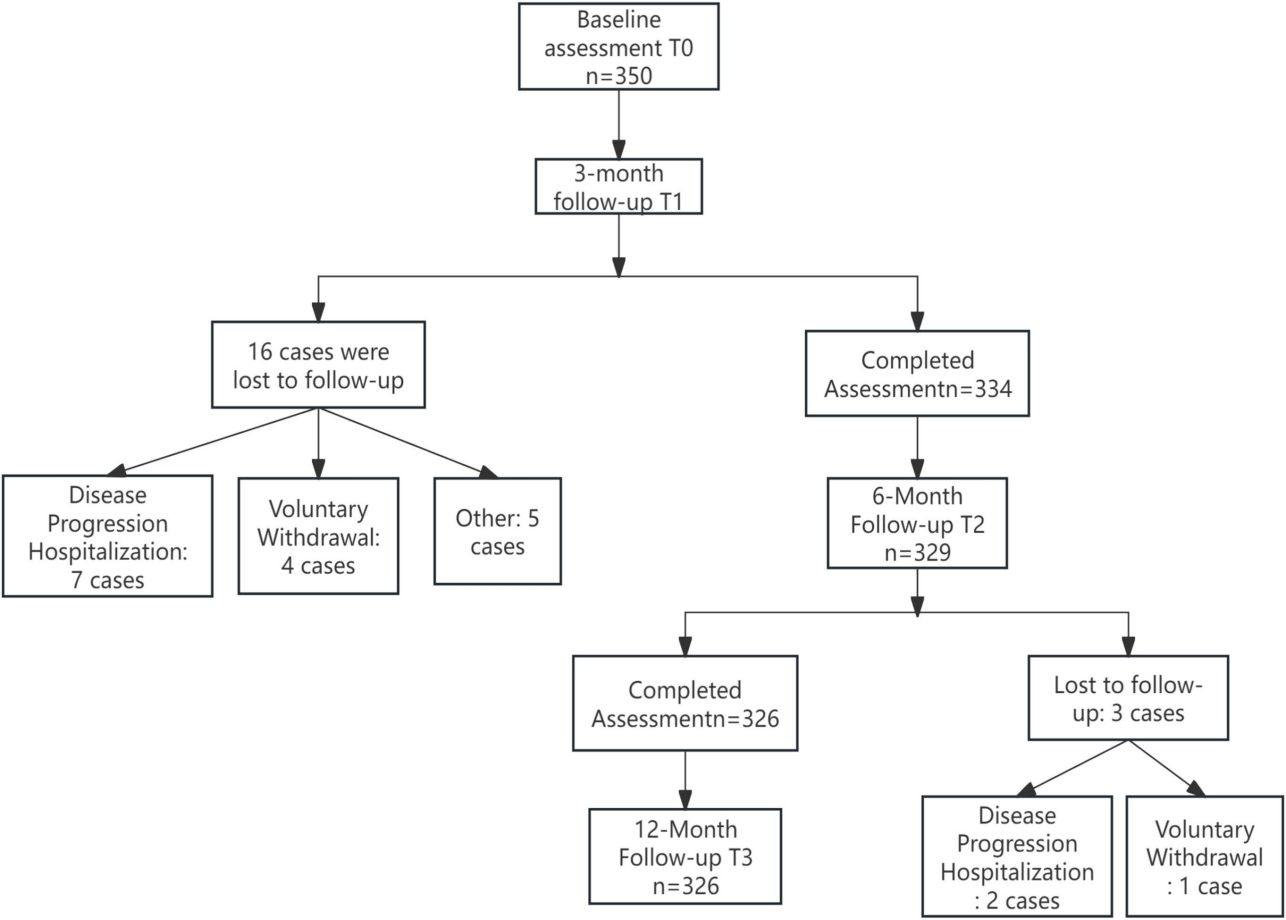
Flowchart of the longitudinal study on burnout and ISI in older adult patients with diabetes and hypertension.

A total of 326 patients were ultimately included in this study. The top two reasons for loss to follow-up were hospitalization due to disease deterioration and voluntary withdrawal, accounting for 71.4% (17 out of 24) of the total loss to follow-up. See Figure 1 for the specific details. The missing mechanism was verified based on the spatiotemporal distribution of the lost-to-follow-up cases as shown in the flowchart. Little’s MCAR test confirmed that the data were completely randomly missing (χ^2^ = 8.32, df = 12, *p* = 0.21). There were no significant differences in core variables such as age (*p* = 0.467) and gender (*p* = 0.31) between the lost-to-follow-up group and the other group. The average age was (68.5 ± 6.2) years, the duration of diabetes was (9.2 ± 4.8) years, the duration of hypertension was (7.8 ± 5.1) years, the baseline glycated hemoglobin was (8.89 ± 2.3)%, the average number of daily medications was (5.3 ± 1.8) types, and the average daily insulin injection volume was (38.2 ± 12.7) units. Other specific details are shown in Table 2.

**Table 2:** General characteristics of the study subjects (*N* = 326).

Variable	Classification	Number	Percentage (%)
Age (years)	60–70	219	67.2
	70~	107	32.8
Gender	Male	150	46.0
	Female	176	54.0
Living pattern	Live by oneself	34	10.4
	Living with spouse/children	292	89.6
Level of education	Primary school and above	135	41.4
	Junior high school	122	37.4
	High school and above	69	21.2
Occupation before retirement	Blue-collar worker	192	58.9
	Brainworker	98	30.1
	Other	36	11.0
Common complications	Coronary heart disease	125	38.3
	Chronic nephrosis	95	29.1
	Peripheral neuropathy	142	43.6
Distribution of monitoring content	Blood Pressure	326	100
	Blood glucose	281	86.2
	Foot	159	48.8
	Weight	113	34.7

### 3.3 Descriptive statistics and correlations of variables

**Figure 2:**
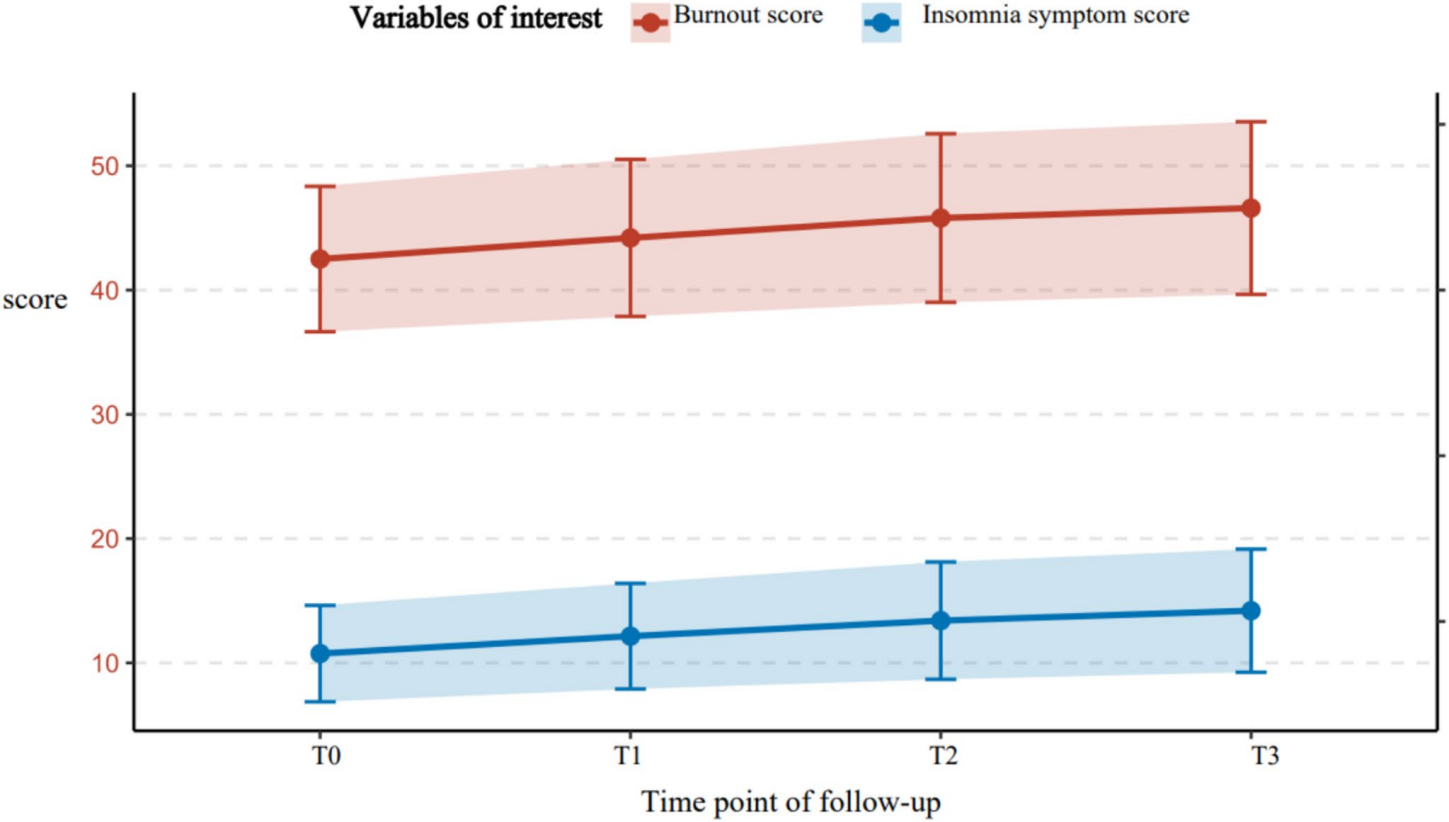
Trajectory of burnout and ISI in older adult patients with diabetes and hypertension.

**Figure 3:**
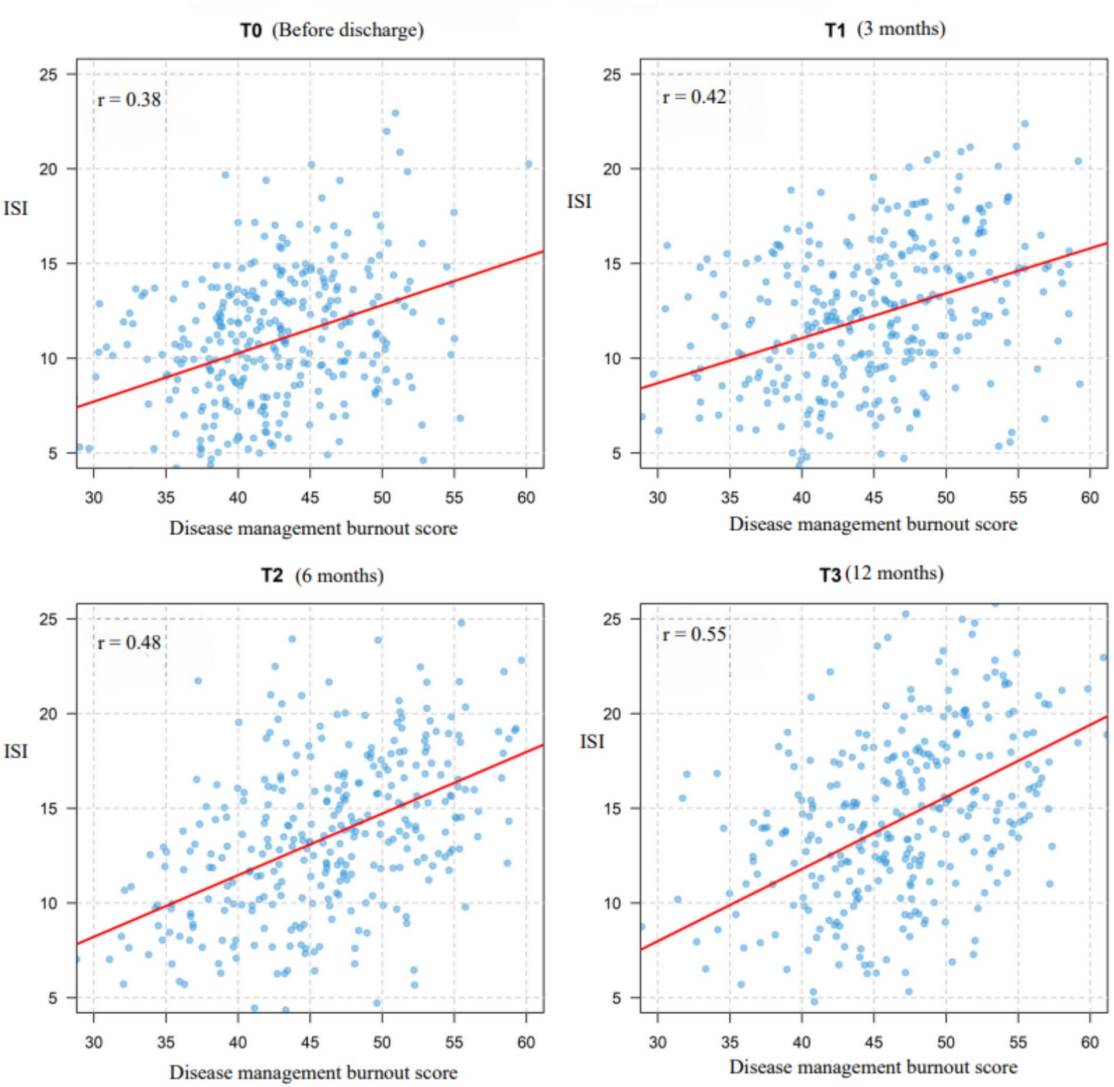
Correlation between disease management burnout and ISI at each time point.

A total of 326 older adult diabetic patients with hypertension were followed up for 1 year, using Huber-White robust standard errors to correct for time-series correlation. All variables exhibited skewness of less than 0.8, meeting the requirements of the linear model. The symptoms of disease management burnout and insomnia demonstrated a continuous worsening trend. Burnout symptoms had a baseline score of 42.50 ± 5.85, which gradually increased over time (T1: 44.20 ± 6.32; T2: 45.80 ± 6.78; T3: 46.60 ± 6.95), with an annual increase of 9.6% (*t* = 10.37, *p* < 0.001). The baseline score for insomnia symptoms was 10.75 ± 3.88, which significantly increased to 14.20 ± 4.95 after 12 months (an increase of 32.1%, *t* = 13.52, *p* < 0.001), indicating progressive deterioration. The strongest correlation was observed at T3 (*r* = 0.55, *p* < 0.001), shown in Figure 2 suggesting that the co-variation intensity of the two symptoms gradually increased with the progression of the disease and peaked at the one-year follow-up. For further details, see Table 3. For related trajectories, see Figure 3.

**Table 3:** Descriptive statistics and correlation analysis of disease management burnout and insomnia symptoms at four time points (*N* = 326).

Variable	x̄ ± s	T0 burnout	T1 burnout	T2 burnout	T3 burnout	T0 ISI	T1 ISI	T2 ISI	T3 ISI
T0Burnout	42.50 ± 5.85	1							
T1Burnout	44.20 ± 6.32	0.41^***^	1						
T2Burnout	45.80 ± 6.78	0.36^***^	0.44^***^	1					
T3Burnout	46.60 ± 6.95	0.31^***^	0.39^***^	0.49^***^	1				
T0 ISI	10.75 ± 3.88	0.38^***^	0.25^**^	0.21^**^	0.17^*^	1			
T1 ISI	12.15 ± 4.25	0.29^***^	0.42^***^	0.28^***^	0.23^**^	0.45^***^	1		
T2 ISI	13.40 ± 4.72	0.24^**^	0.33^***^	0.48^***^	0.30^***^	0.36^***^	0.51^***^	1	
T3 ISI	14.20 ± 4.95	0.19^*^	0.27^***^	0.35^***^	0.55^***^	0.32^***^	0.39^***^	0.58^***^	1

***p < 0.001; **p < 0.01l; *p < 0.05.

### 3.4 Measurement invariance

Longitudinal measurement invariance tests of disease management burnout and insomnia symptoms in older adult patients with diabetes and hypertension were conducted using Mplus 8.7. Based on data from four time points (T0-T3) over a one-year follow-up, two-stage measurement invariance models were established. The first was the configural invariance (configural equivalence) test, which examined whether the factor structure remained consistent across the four time points. The second was the metric invariance (weak invariance) test, which assessed whether the item factor loadings were stable over time. All fit indices met the strict measurement invariance criteria (CFI > 0.97, |ΔCFI| < 0.01), supporting the use of scores from the four time points in the cross-lagged model analysis to ensure the validity of interpreting the time effect. This indicates that the research tool in this study meets the measurement invariance assumption. The specific results are shown in Table 4.

**Table 4:** Results of longitudinal measurement invariance.

Scale	Model	χ^2^	df	CFI	ΔCFI	TLI	RMSEA	ΔRMSEA	SRMR	ΔSRMR
Disease management burnout	Configural Invariance	5208.36	282	0.97	-	0.963	0.04	-	0.03	-
	Metric Invariance	5782.15	298	0.97	−0.002	0.962	0.04	0.001	0.04	0.018
Insomnia symptoms	Configural Invariance	728.41	138	0.98	-	0.982	0.02	-	0.02	-
	Metric Invariance	1035.92	126	0.98	−0.007	0.978	0.02	0.004	0.03	0.012

Configural Invariance: CFI ≥ 0.95, RMSEA ≤ 0.06; Metric Invariance: |ΔCFI| ≤ 0.01, |ΔSRMR| ≤ 0.03.

### 3.5 Model construction and comparison

To assess the longitudinal relationship between disease management burnout and insomnia symptoms, five competing models were constructed, all controlling for covariates such as gender, age, and duration of diabetes. Model 1 included only the first-order autoregressive paths of burnout and insomnia. Model 2 added the cross-time synchronous correlation of burnout → insomnia on the basis of Model 1. Model 3 incorporated the predictive path of disease management burnout → later insomnia, based on Model 2. Model 4 introduced the predictive path of insomnia symptoms → later burnout, based on Model 2. Model 5 integrated all paths from Model 3 and Model 4, including the bidirectional cross-lagged paths of burnout and insomnia. Table 3 indicates that the fit indices of all five models were within acceptable levels (CFI > 0.95, RMSEA < 0.05), but model comparison revealed that Model 5 had the best CFI, RMSEA, and BIC values. Model 5 was selected as the final model, as detailed in Table 5.

**Table 5:** Results of model fitting and comparison.

Model	χ^2^	df	CFI	TLI	RMSEA	SRMR	BIC
Model 1	10492.74	1,044	0.97	0.97	0.03	0.07	1218590.24
Model 2	8752.09	1,042	0.98	0.98	0.02	0.03	1216412.36
Model 3	8701.43	1,040	0.98	0.98	0.02	0.03	1216380.87
Model 4	8703.28	1,040	0.98	0.98	0.02	0.03	1216385.14
Model 5	8658.02	1,038	0.98	0.98	0.02	0.03	1216350.19

Fit criteria: CFI > 0.95, TLI > 0.95, RMSEA < 0.06, SRMR < 0.08.

### 3.6 Cross-lagged analysis of burnout in disease management and insomnia status

**Figure 4:**
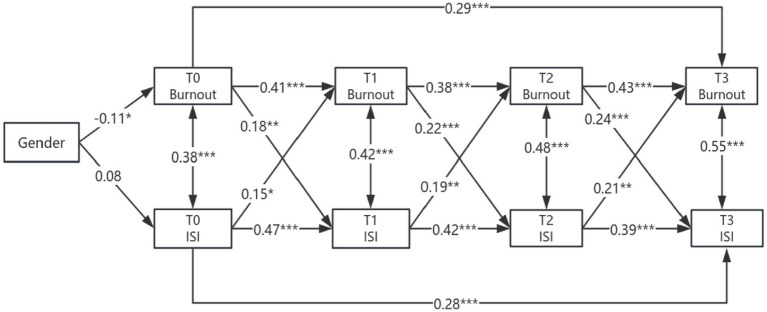
Cross-lagged study of burnout and insomnia in older adult patients with diabetes and hypertension. Gender: Male = 0, Female = 1; ***p < 0.001; **p < 0.01; *p < 0.05; path coefficients are standardized estimates.

The autoregressive paths indicate that (1) there is significant cross-temporal stability between disease management burnout and insomnia symptoms at various time points. The burnout score at T0 positively predicts the scores at T1 and T3, the burnout score at T1 positively predicts the score at T2, and the burnout score at T2 positively predicts the score at T3; the insomnia score at T0 positively predicts the scores at T1 and T3, the insomnia score at T1 positively predicts the burnout score at T2, and the insomnia score at T2 positively predicts the score at T3. (2) The longitudinal paths indicate that the burnout score at T0 positively predicts the insomnia score at T1, the burnout score at T1 positively predicts the insomnia score at T2, and the burnout score at T2 positively predicts the insomnia score at T3; the insomnia score at T0 positively predicts the burnout score at T1, the insomnia score at T1 positively predicts the burnout score at T2, and the insomnia score at T2 positively predicts the burnout score at T3. Show in Figure 4.

### 3.7 The cross-lagged moderating effect of gender in older adult diabetic patients with hypertension

Female patients exhibited significantly higher levels of burnout before discharge (T0; *β* = −0.11, *p* < 0.001). Gender did not significantly influence baseline insomnia (*p* > 0.05), suggesting no disparity in initial sleep status between the genders. There was no gender-based difference in the stability of early burnout (T0 → T1; *p* = 0.18), yet female patients demonstrated greater stability in mid-term burnout (T2 → T3; *β* = 0.45 vs. 0.41, *p* = 0.04). The level of burnout in female patients at T2 strongly predicted insomnia at T3 (*β* = 0.26, *p* < 0.001), with a highly significant intergroup difference (*p* = 0.02), indicating a gender moderation effect. For further details, refer to Table 6.

**Table 6:** The moderating effect of gender in the cross-lagged model.

Influence path	Male group *β* (95% CI)	Female group β (95% CI)	Difference test (*p*)
Direct effect			
Gender → T0 Burnout	-	−0.11 (−0.17, −0.05)^***^	
Gender → T0 Insomnia	-	0.08 (−0.01, 0.17)	0.21
Autoregressive effect			
T0 burnout → T1 burnout	0.40 (0.30, 0.50)^***^	0.42 (0.30, 0.54)^***^	0.18
T2 burnout → T3 burnout	0.41 (0.29, 0.53)^***^	0.45 (0.37, 0.53)^***^	0.04
Cross-lagged moderation			
T2 burnout → T3 insomnia	0.19 (0.13, 0.25)^***^	0.26 (0.16, 0.36)^***^	0.01

***p < 0.001; **indicates that the gender moderation effect is significant when *p* < 0.05 in the difference test. All *β* values in this research report are standardized estimates.

## 4 Discussion

This study, employing a four-time-point cross-lagged model, is the first to reveal the bidirectional vicious cycle mechanism between disease management burnout and insomnia symptoms in elderly patients with diabetes mellitus complicated by hypertension, thereby providing critical empirical support for the behavioral management of metabolic diseases. The core value of the research findings lies in clarifying the unique pathological characteristics of metabolic disease management; by comparing its mechanisms with those of other chronic disease populations, this study deepens the understanding of the association between comorbidity management burden and sleep disorders in chronic diseases.

The observed stepwise increase in burnout and dynamic characteristic of insomnia (rapid exacerbation in the early stage followed by slow progression) essentially reflect the unique “chronic depletion” attribute of metabolic disease management. Burnout in cancer survivors is mostly triggered by acute management burdens such as chemotherapy side effects and adjustments to treatment regimens (19, 20), while in patients with chronic obstructive pulmonary disease (COPD), burnout is often caused by symptom-driven stressors like nocturnal dyspnea and hospitalization due to acute exacerbations (21). In contrast, burnout in patients with diabetes mellitus complicated by hypertension stems from the cumulative effect of daily repetitive management tasks, including blood glucose monitoring, medication compatibility, and dietary control (22, 23). This “chronic depletive burnout” with gradual annual progression is precisely the manifestation of metabolic diseases eroding psychological capital (24); as the disease progresses, the increasing complexity of medication regimens and higher requirements for self-monitoring gradually solidify patients’ sense of loss of control and alienation toward the disease, eventually leading to a stable negative psychological state. The dynamic changes in insomnia symptoms further highlight the pathological specificity of metabolic diseases: the rapid exacerbation of insomnia in the early stage is directly associated with “blood glucose anxiety during the transition period of home-based management,” while the slowed progression in the middle stage is related to hypoglycemia intervention (e.g., insulin dose adjustment) and the elevation of the central fatigue threshold (25, 26). This process stands in stark contrast to the persistent insomnia caused by chemotherapy-induced neurotoxicity in cancer patients (27) and the progressive insomnia resulting from hypoxemia-induced sleep fragmentation in COPD patients (28). Insomnia in metabolic diseases is not driven by a single acute pathological event but rather the outcome of the interaction between “psychological anxiety, metabolic abnormalities, and sleep disorders,” which also explains why the cross-sectional correlation between the two continues to strengthen with disease duration, eventually leading to a phenomenon of synergistic deterioration.

More importantly, the asymmetry of bidirectional effects revealed in this study provides a new perspective for understanding the core contradictions in comorbidity management of chronic diseases: the predictive effect of burnout on subsequent insomnia remains consistently stronger, indicating that management burden is the primary trigger for initiating the vicious cycle in metabolic diseases. This is fundamentally different from the “insomnia preceding burnout” phenomenon observed in the oncology field (e.g., sleep disorders exacerbating treatment-related distress in patients with prostate cancer) (29). and the mechanism in the COPD field where “symptom-driven sleep problems further induce burnout” (i.e., nocturnal dyspnea causes sleep fragmentation, which in turn reduces inhaler adherence) (30). The management of metabolic diseases relies more on patients’ active and sustained engagement—once burnout leads to decreased motivation for self-management (e.g., reduced adherence to blood glucose monitoring), metabolic disorders will further worsen sleep, and insufficient sleep will in turn exacerbate insulin resistance through hypothalamic–pituitary–adrenal (HPA) axis dysfunction, eventually forming a metabolism-specific closed loop of “management slackness → metabolic abnormalities → sleep disorders” (31, 32).

Despite differences in pathological starting points among various chronic diseases, the core pathways of bidirectional interactions between burnout and insomnia can be unified through the biopsychosocial framework. At the biological pathway level, the core trigger for metabolic diseases is the bidirectional feedback between “blood glucose fluctuations and neuroendocrine activation”: asymptomatic nocturnal hypoglycemia directly triggers nocturnal awakenings through sympathetic nerve activation (33), while long-term sleep deprivation exacerbates insulin resistance via HPA axis dysfunction (34). This process shares commonalities with the biological mechanisms of other chronic diseases (all impair central regulatory functions through inflammatory mediators) (35, 36). but also exhibits differences (the inflammatory triggers in metabolic diseases are more routine in daily life). At the psychological pathway level, “a sense of loss of control caused by passive self-management” is the core psychological hub that distinguishes metabolic diseases from other chronic conditions: psychological conflicts in cancer patients mostly stem from death anxiety (37), COPD patients focus on the fear of suffocation (38), while the negative cognition of patients in this study is more inclined to the fixed belief of “treatment ineffectiveness.” When long-term repetitive management tasks fail to yield clear feedback of improvement, patients tend to fall into the cognitive bias of “meaningless effort” (39, 40), and this distortion induces an increase in pre-sleep cortisol levels, forming a self-reinforcing cycle of “cognitive bias → physiological stress → psychological exhaustion” (41). At the social pathway level, “superimposed role burden” is a key inducement for insufficient self-management in metabolic diseases: the core social stress faced by patients is the role conflict between family caregiving and self-management, and elderly patients, in particular, often need to simultaneously undertake tasks such as caring for spouses, housework, and disease management (42); this multi-role pressure leads to decreased management adherence, forming a social closed loop of “social role conflict → insufficient management → disease progression → aggravated burnout.”

The observed gender difference in the female population is essentially the result of the interaction between “biological sensitivity and social roles”: the higher baseline burnout level in female patients originates from the superimposed effect of their dual roles as family caregivers and patients (43), and the long-term management nature of metabolic diseases makes this conflict more persistent. The conversion effect from burnout to insomnia at Time 2 (T2) is stronger in female patients, which is directly associated with the enhanced HPA axis stress sensitivity caused by cyclical fluctuations in estrogen (44). This mechanism shares commonalities with the decreased hypoxic tolerance during hormone fluctuation periods in female COPD patients (45) and the exacerbated emotional disorders due to hormone disturbances after chemotherapy in female cancer patients (46), but the uniqueness of metabolic diseases lies in the interaction between estrogen fluctuations and glucose metabolism (e.g., decreased estrogen exacerbates insulin resistance) (47)—this “hormone-metabolism-psychology” triple interaction makes women more susceptible to the burnout-insomnia cycle.

For the management of elderly patients with hypertension complicated by diabetes, clinical practice can block the burnout→insomnia vicious cycle from three aspects: during the high-risk middle stage of disease management, real-time tracking of nocturnal hypoglycemic events via continuous glucose monitoring (48), and delivery of coping strategies through intelligent reminder systems can reduce the risk of sleep fragmentation; developing personalized management courses centered on motivational interviewing can enhance self-management efficacy; adopting multi-modal sleep interventions (e.g., cognitive behavioral therapy for insomnia combined with mindfulness training) can correct sleep catastrophizing cognition (49); and combining anti-inflammatory drugs or nutritional supplements can alleviate prefrontal lobe function impairment (50, 51). For female patients, it is necessary to establish a collaborative support network integrating family, community, and medical institutions to share caregiving pressure, design stage-specific psychological adjustment programs based on the estrogen fluctuation cycle, and strengthen neuroendocrine regulatory interventions during periods of hormone level fluctuation to reduce the risk of burnout conversion.

Limitations of This Study This study has several limitations. First, although structural equation modeling clarified the longitudinal bidirectional relationship between disease management burnout and insomnia symptoms, the statistical control of potential confounding variables was not exhaustive. Due to data availability constraints, the study failed to systematically assess and adjust for the severity of comorbidities, specific medication types (e.g., those known to affect sleep or mood), nor did it conduct standardized screening for anxiety/depression symptoms using tools such as the GAD-7 or PHQ-9. These unmeasured confounders may affect the accuracy and interpretability of the estimated path coefficients (e.g., *β* values). Future research should incorporate these variables to more accurately clarify the net effects between the core variables. Second, the study only used subjective sleep assessment indicators and lacked objective verification via polysomnography or actigraphy. This limitation was particularly prominent when assessing sleep fragmentation patterns (e.g., awakenings caused by nocturnal hypoglycemia), ultimately restricting measurement precision. Finally, the single-center sampling design and underrepresentation of rural patients limited the generalizability of the study results to populations with inadequate access to medical services. Future research should prioritize multicenter cohort validation to improve external validity and integrate standardized mental health assessment tools and objective sleep monitoring methods to address the aforementioned limitations of this study.

## 5 Conclusion

This one-year longitudinal study identified a bidirectional vicious cycle between disease management burnout and insomnia symptoms in elderly patients with diabetes complicated by hypertension. The 6-month post-discharge period was found to be a critical window for clinical intervention. These findings elucidate the behavioral-sleep comorbidity mechanism in chronic disease management and establish a theoretical foundation for integrated sleep and self-management interventions.

## Data Availability

The original contributions presented in the study are included in the article/supplementary material, further inquiries can be directed to the corresponding author.
